# Enhanced Angiogenesis in Salivary Duct Carcinoma Ex-Pleomorphic Adenoma

**DOI:** 10.3389/fonc.2020.603717

**Published:** 2021-02-22

**Authors:** Takayoshi Suzuki, Satoshi Kano, Masanobu Suzuki, Shinichiro Yasukawa, Takatsugu Mizumachi, Nayuta Tsushima, Kanako C. Hatanaka, Yutaka Hatanaka, Yoshihiro Matsuno, Akihiro Homma

**Affiliations:** ^1^Department of Otolaryngology-Head and Neck Surgery, Graduate School of Medicine, Hokkaido University, Sapporo, Japan; ^2^Clinical Research & Medical Innovation Center, Hokkaido University Hospital, Sapporo, Japan; ^3^Department of Surgical Pathology, Hokkaido University Hospital, Sapporo, Japan

**Keywords:** salivary duct carcinoma, carcinoma ex-pleomorphic adenoma, VEGFA, HER2, machine learning

## Abstract

Salivary duct carcinoma (SDC) is morphologically similar to breast cancer, with HER2-overexpression reported. With regard to the pattern of disease onset, SDC can arise from *de novo* or carcinoma ex-pleomorphic adenoma (Ca-ex-PA). Recently, multiple molecular profiles of SDC as well as breast cancer have been reported, with significant differences in HER2 expression between Ca-ex-PA and *de novo*. We assessed the differences in gene expression between onset classifications. We conducted immunohistochemical analysis and HER2-DISH for 23 patients and classified SDCs into three subtypes as follows: “HER2-positive” (HER2+/any AR), “Luminal-AR” (HER2-/AR+), and “Basal-like” (HER2-/AR-). We assessed the expression levels of 84 functional genes for 19 patients by using a qRT-PCR array. Ten cases were classified as HER2-positive, seven cases as Luminal-AR, and six cases as Basal-like. The gene expression pattern was generally consistent with the corresponding immunostaining classification. The expression levels of VEGFA, ERBB2(HER2), IGF1R, RB1, and XBP1 were higher, while those of SLIT2 and PTEN were lower in Ca-ex-PA than in *de novo*. The functions of those genes were concentrated in angiogenesis and AKT/PI3K signaling pathway (Fisher’s test: p-value = 0.025 and 0.004, respectively). Multiple machine learning methods, OPLS-DA, LASSO, and RandomForest, also show that VEGFA can be a candidate for the characteristic differences between Ca-ex-PA and *de novo*. In conclusion, the AKT/PI3K signaling pathway leading to angiogenesis was hyper-activated in all SDCs, particularly in those classified into the Ca-ex-PAs. VEGFA was over-expressed significantly in the Ca-ex-PA, which can be a crucial factor in the malignant conversion to SDC.

## Introduction

Salivary duct carcinoma (SDC) is a highly aggressive type of carcinoma, similar to breast cancer morphologically (1, 2). Recently, the histological resemblance of SDC to breast cancers has led to the study of HER2 (human epidermal growth factor receptor 2, also known as ERBB2) expression (3). HER2 overexpression or amplification is seen in 15–20% of patients with invasive breast cancers and is considered to be an adverse prognostic factor (4). Strong immunohistochemical staining for HER2 protein has also been identified in 25–92% of SDCs (5). These findings highlight the similarity between SDC and breast cancer with regard not only to the overall morphology but also the immunophenotype and gene expression profile. Moreover, a few investigators have reported that the treatment regimens, including the use of HER2 antagonists, result in variable clinical benefits to patients with HER2-positive SDCs, although these protocols are not necessarily effective for every patient with SDC (6–11). These results indicate that the concept of “SDC”, as well as that of breast cancer, should not be regarded as a single disease but as a collection of heterogeneous entities with various characteristics. Based on the molecular biological profile, breast cancers can be stratified into multiple subtypes and individualized treatments are indicated for each subtype. In practice, anti-HER2 antibodies have already been applied for HER2-positive breast cancers, with clinical benefits observed in metastatic and adjuvant settings (12, 13). In SDCs as well, individualized treatment selection is expected to be based on stratification (14, 15).

Although a few clinical trials from Japan, such as combined androgen blockade for AR (Androgen receptor)-positive salivary gland cancer and trastuzumab, an anti-HER2 antibody, plus docetaxel therapy for HER2-positive SDCs, have brought favorable results (11, 16), there is no stratification currently available for personalized treatment selection and no consensus regarding a standard treatment or protocol. For the development of therapeutic methods with clinical applications, further understanding of SDC tumorigenesis is necessary.

Moreover, it is well-known that SDC, as well as other salivary gland cancers, can occur *de novo* or as a malignant component of carcinoma ex-pleomorphic adenoma (Ca-ex-PA) (17). For this reason, we focus on the relationship between the biological profile and onset pattern (*de novo* or Ca-ex-PA) of SDC based on the hypothesis that SDCs have different biological profiles depending on whether they arise *de novo* or Ca-ex-PA. This study aims to clarify and explore the etiology and onset mechanism of SDCs. We examined their gene expression profiles and immunohistology, and compared these between Ca-ex-PA and *de novo* as well as among classifications based on their immunohistological profiles.

In particular, we evaluated the following;

1. Classification into immunostaining-status-based subtypes as follows: “HER2-positive” (HER2+/any AR), “Luminal-AR” (HER2-/AR+), and “Basal-like” (HER2-/AR-), (Di Palma classification) (18).
2. Relationship between Di Palma classification and gene expression.
3. Relationship between onset classification (*de novo* vs. Ca-ex-PA) and Di Palma classification.
4. Relationship between onset classification (*de novo* vs. Ca-ex-PA) and gene expression levels by using a two-group comparison test and multiple machine learning methods.

## Materials and Methods

### Patient Selection and Histological Review

We retrospectively analyzed 23 patients with untreated SDCs, who underwent surgery as a primary treatment in Hokkaido University Hospital, Japan, between 1991 and 2015. All tumors were confirmed to have been diagnosed accurately by two expert pathologists (TS and KH) according to the rigorous histomorphologic criteria for SDC (2). We conducted a histological review of the multi-step sections from the entire tumor in each case to classify SDCs into Ca-ex-PA group and *de novo* in accordance with the current WHO classification. All patients were treated with surgery as a primary treatment, and most underwent subsequent postoperative irradiation and/or chemotherapy.

### Tissue Microarray

Tissue microarray blocks were constructed using a manual tissue microarrayer (JF-4; Sakura Finetek Japan, Tokyo, Japan) with a 1.5 mm diameter needle. The finalized blocks were sliced into 4 mm-thick sections and mounted on glass slides. To check the histopathological diagnosis and adequacy of tissue sampling, a section from each microarray was stained with hematoxylin and eosin and examined by two expert pathologists (TS and KH).

### Immunohistochemistry

For immunohistochemistry (IHC), a polymer-based detection system with heat-mediated antigen retrieval was employed. Diaminobenzidine was applied to detect antigen-antibody reactions. Appropriate positive and negative controls were employed for all conditions. IHC for HER2 (4B5, Ready-To-Use, Ventana) and AR (AR27, 1:50 dilution, Leica) was performed according to the respective manufacturer’s recommendations.

### HER2/CEN17 Dual Color *in situ* Hybridization

Dual color *in situ* hybridization (DISH) analysis using a Benchmark ULTRA system (Ventana Medical Systems, CA) was carried out for all 23 SDC cases. A 4 μm-thick paraffin-embedded tumor tissue microarray was placed onto a glass slide and subjected to DISH. HER2 amplification was performed in accordance with the manufacturer’s instructions using DISH HER2 PharmDx (Dako, Glostrup, Denmark). Both HER2 signaling (black signal) and the chromosome 17 centromere (CEN17) (red signal) were depicted and counted to calculate the ratio of the total number of HER2 signals to the total number of CEN17 signals.

### Scoring System for Immunostaining Classification

For HER2, the ASCO/CAP scoring system was used as follows: Negative, no membrane staining or <10% of cells stained; 1+, incomplete membrane staining in >10% of cells; 2+, >10% of cells with weak to moderate complete membrane staining; and 3+, strong and complete membrane staining in >30% of cells (19). Cases were considered HER2-positive if HER2 staining was scored as 3+ or 2+ with HER2 gene amplification as defined by in-situ hybridization.

AR expression level was semi-quantitatively counted every 10 percent. Nuclear staining was evaluated as positive. With regard to AR scoring, we considered a nuclear positivity ≥1% as positive according to ASCO/CAP 2013.

### Classification Into Immunostaining Status-Based Subtypes (Di Palma Classification).

In accordance with the classification proposed by Di Palma et al., we classified the 23 cases with SDC into three subtypes as follows: “HER2-positive” (HER2+/any AR), “Luminal-AR” (HER2-/AR+), and “Basal-like” (HER2-/AR-) (18).

### PCR Array for Gene Expression

RT2 Profiler PCR Arrays^®^ are tools for analyzing the expression of a focused panel of genes. Each 96-well plate PCR array includes SYBR^®^ Green-optimized primer assays for a thoroughly researched panel of relevant, pathway- or disease-focused genes simultaneously under uniform cycling conditions. Total RNA was isolated using a FFPE Kit (QIAGEN, #217504). cDNA was synthesized using RT2 SYBR Green ROX qPCR MasterMix (QIAGEN, #330522). Four of the 23 samples were excluded due to the low quality of the RNA. The Human Breast Cancer RT^2^Profiler™ PCR Array (QIAGEN, PAHS-131ZC-12) was used for the reaction in accordance with the manufacturer’s instructions. Amplification and real-time analysis were performed with a StepOnePlusTM real-time PCR system (Thermo Fisher Scientific, Waltham, MA, USA). Transcript levels were normalized against the *GAPDH* (Glyceraldehyde-3-Phosphate Dehydrogenase) RNA levels. The relative mRNA expression levels were calculated according to the comparative Ct (ΔΔCt) method. We excluded six genes, including *ADAM23* (ADAM Metallopeptidase Domain 23), *BIRC5* (Baculoviral IAP Repeat Containing 5), *BRCA2* (Breast cancer 2, early onset), *CCNA1* (Cyclin A1), *RARB* (Retinoic Acid Receptor, Beta), and *TWIST1* [Twist homolog 1 (Drosophila)], from the analysis as more than half of the values were missing. The function of each gene was referred to the document included with the PCR Array kit (**Table S1**). All error bars represent the standard error value across biological replicates divided by the square root of the sample size (SEM). Where technical replicates were conducted, these values were averaged to yield a single value per biological replicate and, subsequently, all data are shown as means ± SEM.

### Relationship Between Onset Classification (*de novo* vs. Ca-ex-PA) and Gene Expression by Using a Two-Group Comparison Test and Multiple Machine Learning Methods

To compare gene expression profiles between Ca-ex-PA and *de novo*, we performed a two-group comparison test on 78 genes for 19 cases. Subsequently, we searched for characteristic gene expression patterns between the onset classifications by using multiple machine learning methods; Orthogonal projections to latent structures discriminant analysis (OPLS-DA), LASSO, and RandomForest (RF).

OPLS-DA is a supervised machine learning method that employs a linear multivariate discriminant model, following projection of the predicted variables and observable variables to a new space (20–22). OPLS-DA can clarify the difference between two groups of high-dimensional data. Practically, by generating a principal component that maximizes the distance between the centroid of two groups and quantifying the weight of the principal component as the importance of the factor contributing to classification (VIP: Variable Influence on Projection), we can clarify the characteristic differences between two groups in an interpretable manner (21, 23–25). Furthermore, this method can avoid multicollinearity issues, which can be often assumed due to the large number of genes relative to that of samples in our study. We performed OPLS-DA with 6-fold cross-validation and 10,000 permutations to explore gene importance as a classifier for onset classification and clarify the mechanism for disease onset. The first principal component of variable importance in the projection (VIP) value above 1.5 is taken as a significant value for classification, suggesting the genes that characterize Ca-ex-PA or *de novo*.

LASSO is a logistic regression analysis method with L1 regularization in order to enhance the prediction accuracy and interpretability of the statistical model it produces (26). It sets the coefficients of less significant variables to 0 by assigning a L1-penalty (lambda) to regression coefficients, and only more significant variables are extracted. A leave-one-out cross-validation (LOOCV) with the grid-search method is used to determine the parameter lambda for optimization of the area under the ROC Curve (AUC).

RF is an ensemble learning method for classification or regression that functions by constructing a multitude of decision trees during training and outputting the mode of the classes (classification) or the mean prediction (regression) of the individual trees (27). RF is able to deal with high-dimensional data in a nonparametric manner, which allows us to assess interactive and nonlinear (regression) effects. Recently, feature selection based on the random forest classifier has been found to provide multivariate feature importance scores that are relatively effective, and which have been successfully applied to high-dimensional data arising from microarrays (28).

We performed RF analysis for onset classification to explore gene importance as a classifier for onset classification and to clarify the mechanism underlying disease onset. In accordance with the default settings of the R package “RandomForest”, the number of variables used per decision tree was set at eight. To build a CART model, 13 out of the 19 samples were selected to allow duplication. The number of decision trees was set at 10,000. This final prediction is determined by the principle of majority vote. We adopted MeanDecreaseGini as the variable importance index for classification.

### MissForest

Biomedical research based on high-throughput technology often faces the problem of missing data. Algorithms commonly used in the analysis of such large-scale data often depend on a complete set, particularly for some machine learning methods.

Multiple imputation (MI) has been widely used for handling missing data in biomedical research (29). The most prevalent multiple imputation methods, such as k-nearest-neighbors for continuous data, saturated multinomial model for categorical data and multivariate imputation by chained equations for mixed data types, depend on tuning parameters or specification of a parametric model such as a linear data structure; however, real-world data does not necessarily follow these assumptions (30–32).

MissForest is an iterative imputation method based on a random forest, by averaging over many unpruned classification or regression trees. The characteristics of the non-parametric and randomized method in this algorithm can be applied to real-world data without strict assumptions about the distributional aspects of the data. Practically, it is also known to achieve better performance without tuning parameters.

We impute the missing values for our “gene expression” data including missing values with the R package “missForest” (33). The percentage of missing values was 9.8% (145/1,482), which suggests that imputation by “missForest” is applicable from the viewpoint of imputation quality (34). In accordance with the default settings of the R package “missForest”, the number of decision trees was set at 100, and the number of variables used per decision tree was set at eight. We calculated the OOB imputation error rate based on the normalized root mean squared error and evaluated the complement accuracy. The OBB error rate value converged to around 0.483. These imputed data were then applied to OPLS-DA, LASSO, and RandomForest.

### Partitioning the Dataset for Machine Learning

Supervised machine learning methods typically require the partitioning of data into training data and test data. According to the “70-30 rule”, we randomly split the data into 70% for training and 30% for test with the R package “caret”. These training data and test data were then applied to LASSO.

### Statistical Analysis

All statistical analyses were performed using the software program R ver. 3.5.1 (R Foundation for Statistical Computing, Vienna, Austria. URL https://www.R-project.org/.). We used R packages “caret”, “glmnet”, “pROC”, “missForest”, “randomForest”, and “ranger” in this analysis.

## Results

### Classification Into Immunostaining Status-Based Subtypes (Di Palma Classification).

**Figure 1:**
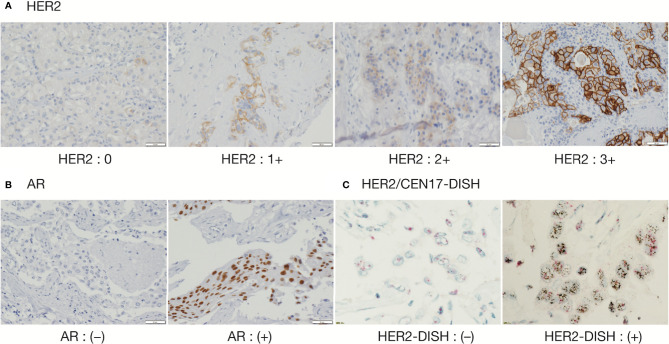
Immunohistochemical staining of HER2 and AR, and Dual Color in Situ Hybridization (DISH) of HER2/CEN17. **(A)** HER2 (magnification, 200 ×), **(B)** AR (magnification, 200 ×), and **(C)** HER2/CEN17-DISH (magnification, 200 ×).

IHC and DISH images are shown in **Figure 1**. For HER2-IHC, we classified 10 cases (43.5%) as 3+, 5 cases (21.7%) as 2+, 1 case (4.3%) as 1+, and 7 cases (30.4%) as 0. For HER2-DISH, nine cases (40.9%) were found to be positive, with the HER2-IHC score of all nine cases being 3+. Only one case showed a HER2-IHC score of 0, and no HER2-DISH assessment was possible as no CEN17 signal was observed for the slide. Finally, 10 SDCs were evaluated as HER2-positive (**Table 1**).

**Table 1:** HER2-IHC-DISH cross table.

	HER2-IHC			
HER2-DISH	3+	2+	1+	0
**positive**	9	0	0	0
**negative**	1	5	1	6

The proportion of AR-positive cells showed a bimodal distribution and the median percentage of AR-positive cells was 80% (1st-quantile: 20.0, 3rd-quantile: 85.0, IQR: 65.0). Seventeen of 23 cases (73.9%) were considered to be AR-positive (**Table 2**).

**Table 2:** HER2-AR cross table.

	AR-IHC	
HER2-score	positive	negative
**positive**	10	0
**negative**	7	6

In the current study, 10 cases were classified as HER2-positive, seven cases as Luminal-AR, and six cases as Basal-like (**Table 3**).

**Table 3:** The Di Palma Classification.

	HER2	AR	Cases
**HER2-positive**	+	+/−	10
**Luminal-AR**	−	+	7
**Basal-like**	−	−	6

### Relationship Between Di Palma Classification and Gene Expression

**Figure 2:**
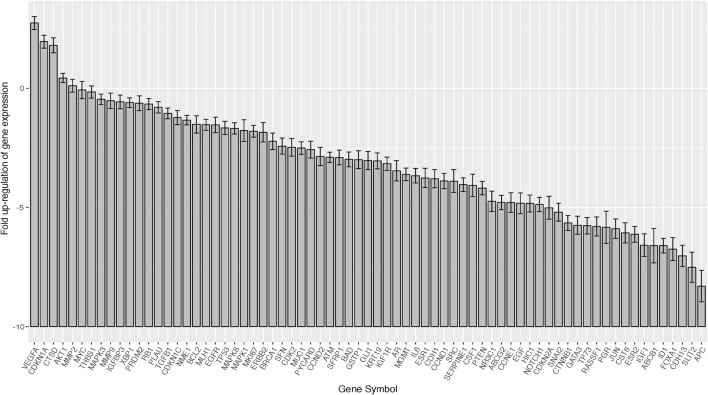
Barplots of gene expression in salivary duct carcinomas (19 samples × 78 genes). In the total SDC group, VEGFA, AKT1, MMP2, MAPK3, MMP9, and IGF1R were relatively over-expressed, while SLIT2 was suppressed. Error bar means ±2×SEM (standard error of the mean).

We assessed the expression of 78 gene for 19 cases, including 10 HER2-positive, 5 Luminal-AR, and 4 Basal-like cases, by using a quantitative RT-PCR array (**Figure 2**).

To evaluate whether Di Palma classification reflects the gene expression status, we assessed 14 cancer classification marker genes corresponding to immunostaining status: “HER2-positive Cancer”: *ERBB2* and *GRB7* (Growth factor receptor-bound protein 7), “Luminal-AR Cancer”: *AR*, *ESR1* (Estrogen receptor 1), *FOXA1* (Forkhead box A1), *GATA3* (GATA binding protein 3), *KRT8* (Keratin 8), *KRT18* (Keratin 18), *SLC39A6* (Solute carrier family 39), *TFF3* (Trefoil factor 3), and *XBP1* (X-box binding protein 1), and “Basal-Like Cancer”: *EGFR*, *KRT5* (Keratin 5), and *NOTCH1* (Notch 1). Among these genes, nine genes (*ERBB2*, *GRB7*, *AR*, *ESR1*, *GATA3*, *KRT18*, *SLC39A6*, and *TFF3)* were found to have significant differences in expression according to Di Palma classification, with the gene expression patterns showing significant differences being generally consistent with their markers for immunostaining classification (**Table 4**). Except for those 14 cancer classification marker genes, there were significant differences in the expression of three genes: *GLI1* (GLI family zinc finger 1), *KRT19* (Keratin 19), and *VEGFA*.

**Table 4:** “Subtyping marker” gene expression among Di Palma classification.

Classification marker	Statistical test	p-value in subtypes	Post-hoc	p-value in H vs B	p-value in L vs B	p-value in L vs H	Significant status of gene expression
**HER2-positive**							
HER2	Oneway-ANOVA	**< 0.001**	TukeyHSD	**0.003**	0.822	**0.002**	H > B, H > L
GRB7	Oneway-ANOVA	**0.011**	TukeyHSD	**0.049**	0.961	**0.027**	H > B, H > L
**Luminal-AR**							
AR	Oneway-ANOVA	**0.022**	TukeyHSD	**0.018**	0.152	0.789	H > B
ESR1	Oneway-ANOVA	**0.040**	TukeyHSD	0.568	**0.041**	0.107	L > B
FOXA1	Kruskal-Wallis	0.302					
GATA3	Oneway-ANOVA	**0.042**	TukeyHSD	0.422	**0.037**	0.154	L > B
KRT8	Oneway-ANOVA	0.087					
KRT18	Oneway-ANOVA	**0.013**	TukeyHSD	0.065	**0.010**	0.325	L > B
SLC39A6	Oneway-ANOVA	**0.019**	TukeyHSD	0.188	**0.015**	0.162	L > B
TFF3	Oneway-ANOVA	**0.036**	TukeyHSD	0.690	**0.043**	0.076	L > B
XBP1	Oneway-ANOVA	**0.023**	TukeyHSD	0.083	0.985	**0.040**	H > B, H > L
**Basal-like**							
EGFR	Kruskal-Wallis	0.433					
KRT5	Oneway-ANOVA	0.523					
NOTCH1	Kruskal-Wallis	0.225					

Bold letters were applied to emphasize for p-values less than 0.05.

### Relationship Between Onset Classification (*de novo* vs. Ca-ex-PA) and Di Palma Classification

Of the 14 cases of Ca-ex-PA, nine were classified as “HER2-positive”, three as “Luminal-AR”, and two as “Basal-like”. On the other hand, of the nine cases of *de novo* SDC, one was classified as “HER2-positive”, four as “Luminal-AR”, and four as “Basal-like”. A comparison of *de novo* and Ca-ex-PA revealed more frequent HER2-positivity in Ca-ex-PA than in *de novo* (Fisher’s exact test: p-value = 0.029) (**Table 5**).

**Table 5:** Comparison between the Di Palma classification and onset classification.

	Ca-ex-PA type	de novo type
**HER2-positive**	9	1
**Luminal-AR**	3	4
**Basal-like**	2	4

### Relationship Between Onset Classification (*de novo* vs. Ca-ex-PA) and Gene Expression by Using a Two-Group Comparison Test and Multiple Machine Learning Methods

1. Two-Group Comparison Test

**Figure 3:**
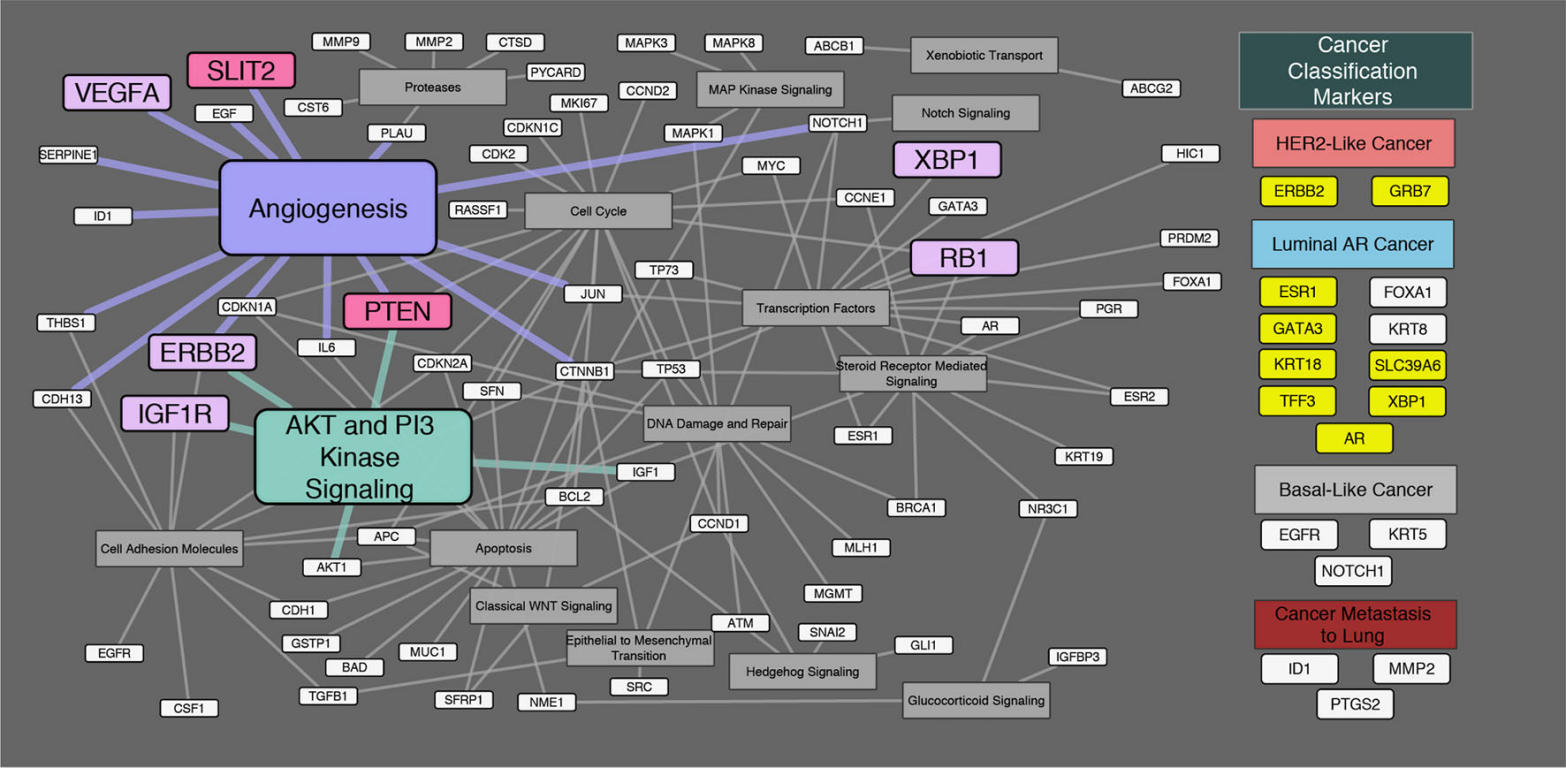
Diagram of the relationship between genes and their genetic functions. Correspondence between each gene and its function is shown by connections between lines. In the “Cancer Classification Markers” section, 17 classification marker genes were categorized in accordance with Di Palma classification. Filled “yellow” highlights represent significant differences in gene expression by immunostaining classification. Filled “violet” highlights represent significant differences in gene expression by onset classification (Ca-ex-PA > *de novo*). Filled “pink” highlights represent significant differences in gene expression by onset classification (*de novo* > Ca-ex-PA). Analysis by genetic function revealed that the significant differences in expression between Ca-ex-PA and *de novo* were concentrated in genes associated with angiogenesis and the AKT/PI3K signaling pathway (Fisher’s test: p-value = 0.025 and 0.004, respectively).

Regarding onset classification, seven genes showed significant differences in gene expression between Ca-ex-PA and *de novo*: *VEGFA*, *ERBB2*, *IGF1R* (Insulin-like growth factor 1 receptor), *RB1* (Retinoblastoma 1), *XBP1*, *SLIT2*, and *PTEN* (Phosphatase and tensin homolog). In Ca-ex-PA type SDCs, the gene expression of *VEGFA*, *ERBB2*, *IGF1R*, *RB1*, and *XBP1* was observed to increase, while that of *SLIT2* and *PTEN* decreased. Analysis by genetic function showed that the significant differences in gene expression between Ca-ex-PA and *de novo* were concentrated in genes associated with angiogenesis and the AKT/PI3K signaling pathway (Fisher’s test: p-value = 0.025 and 0.004, respectively) (**Table 6**, **Figure 3**).

**Table 6:** Gene expression by onset classification.

Gene Symbol	Statistical test method	p-value in subtype	Features of gene expression patterns
***HER2***	student’s T	**0.030**	Ca-ex-PA > de novo
***IGF1R***	student’s T	**0.043**	Ca-ex-PA > de novo
***PTEN***	student’s T	**0.044**	de novo > Ca-ex-PA
***RB1***	student’s T	**0.026**	Ca-ex-PA > de novo
***SLIT2***	student’s T	**0.046**	de novo > Ca-ex-PA
***VEGFA***	student’s T	**0.034**	Ca-ex-PA > de novo
***XBP1***	student’s T	**0.037**	Ca-ex-PA > de novo

Bold letters were applied to emphasize for p-values less than 0.05.

2. Orthogonal Projections to Latent Structures Discriminant Analysis

After 10,000 permutation tests, the pQ2 value was 0.0352, suggesting that the OPLS-DA model had not been over-fitted, and the two groups had significant differences in terms of OPLS-DA score maps (spectral separation) (**Figure S1**). In this OPLS-DA model, 14 genes of VIP >1.5 were found, ordered as follows: *VEGFA*, *XBP1*, *PTEN*, *PYCARD*, *TFF3*, *MKI67*, *RASSF1*, *TP73*, *SLIT2*, *CDK2*, *ERBB2*, *ESR2*, *MMP2*, and *BRCA1*. *VEGFA* and *PTEN* had high values of VIP, which were considered to be related to angiogenesis (**Figure S2**).

3. LASSO

Nine genes were extracted as sufficiently significant, in the following order: *MKI67*, *AR*, *GSTP1*, *CTSD*, *PTEN*, *ID1*, *SFRP1*, *CSF1*, and *KRT18* (**Figure S3**). The sensitivity, specificity, and AUC were 0.6667, 1.0000, and 0.8333, respectively.

4. RandomForest

*NOTCH1, CDKN1C, ID1, KRT8, RB1*, and *VEGFA* had high MeanDecreaseGini values for onset classification, most of which genes are considered to be related to angiogenesis (**Figure S4**). We calculated the Out-Of-Bag (OOB) rate for an estimate of the generalization error, whose value converged to around 0.368 for onset classification.

## Discussion

Salivary duct carcinoma (SDC) was first described as morphologically similar to breast cancers in 1968 (1). SDC presents as a rapidly growing mass with the potential for local recurrence and neck and/or distant metastases. The current standard treatment for SDC is complete surgical resection with lymph node dissection and adjuvant radiotherapy. However, the results of standard therapy continue to be disappointing. More than 50% of patients die of disease within 3 to 5 years despite aggressive surgical resection and radiotherapy, and the overall 5-year survival rate is 42–55% (35–39). Moreover, therapeutic options for patients with advanced unresectable primary, recurrent, or metastatic disease are particularly limited (40).

In breast cancer, therapeutic strategies based on clinicopathology and molecular biology have already been established, and these have contributed to our understanding of the developmental mechanisms of the disease and have been applied in clinical settings. In SDC, on the other hand, although a new classification based on immune-profiles has been proposed by Di Palma et al. (18), there have been few studies to date on whether the classification reflects the molecular biological status.

Subsequently, we focused on onset classification and analyzed the differences in gene expression between Ca-ex-PA and *de novo* SDCs. As shown in **Table 6**, different gene profiles were observed for the two groups, with seven genes showing significant differences in gene expression. Interestingly, their functions were generally associated with angiogenesis and the AKT/PI3K signaling pathway (**Figure 3**).

### Di Palma Classification Represents Gene Expression Profiles, and Subtypes Have Different Gene Expression Profiles

Previous articles have reported that approximately 80 to 90% of patients with SDCs are positive for AR, and 30 to 40% are positive for HER2, which is similar to our results (2, 14, 41–46). Thus, the patients in this study are thought to afford a similar population to those of the previous articles.

We examined whether Di Palma classification, based on immunostaining status, can be applied to gene expression profiles by comparing the immunostaining status with the expression of the corresponding gene. Among 14 cancer classification marker genes corresponding to immunostaining status, nine genes were found to show significant differences in gene expression among Di Palma classifications, suggesting that immunostaining appropriately reflects the gene expression profiles and that each subtype based on Di Palma classification (“HER2-positive”, “Luminal-AR”, and “Basal-like”), has a different gene expression profile.

### Characteristic Differences Between Ca-Ex-PA and *de novo*

In the current study, HER2-positive cases were observed more frequently in the Ca-ex-PA than in the *de novo* (Fisher’s exact test: p-value = 0.029), suggesting that there is a tendency for characteristic immunostaining differences to exist between the two types of SDC.

Subsequently, we searched for characteristic differences in gene expression between the onset classifications. First, we compared the gene expression on 78 genes for 19 cases between Ca-ex-PA and *de novo* with a two-group comparison test.

Next, we wanted to perform multivariate analysis to remove the influence of confounding factors; however, the number of genes evaluated for the number of cases was so large that it was considered likely to result in a problem of multiple collinearity. Therefore, we applied a number of machine learning methods to search for characteristic differences in gene expression between the onset classifications.

As a result, the two-group comparison test and machine learning methods showed that genes related to angiogenesis could be extracted as candidates displaying characteristic differences between Ca-ex-PA and *de novo*. In particular, VEGFA, which plays a primary role in angiogenesis, was selected as a candidate to explain the characteristic differences between Ca-ex-PA and *de novo* in all assays.

### Angiogenesis in Salivary Duct Carcinoma

In the current study, *VEGFA* was over-expressed in SDCs on the whole, and particularly in the SDCs in the Ca-ex-PA group. *VEGFA* is a member of the VEGF family of genes, which are particularly important to the induction of angiogenesis (47). *VEGF* has been reported to be expressed at high levels in most cancers (48), and to be associated with increased risk of recurrence, metastasis, and death in NSCLCs and RCCs (49–51).

Faur et al. investigated whether salivary gland tumors with a different morphology and evolution also differ in terms of neo-vascularization and VEGF expression, and the prognostic value of the results (52). Surgical specimens (8 PAs, 7 Warthin tumors, 5 basal cell adenomas, 6 Ca-ex-PAs, 6 mucoepidermoid carcinomas, 5 acinic cell carcinomas, 4 adenoid cystic carcinomas, and 4 adenocarcinomas not otherwise specified) were immune-stained. Malignant salivary gland tumors showed a significantly higher level of VEGF expression compared to benign tumors (p = 0.001). Fonseca et al. reported VEGF immunostaining for 132 salivary gland tumors (50 PAs, 32 mucoepidermoid carcinomas, 30 adenocarcinomas not otherwise specified, and 20 adenoid cystic carcinomas) and its relationship with their histopathological type (53). VEGF expression was found in the cytoplasm in all cases, proving to be overexpressed in malignant tumors in comparison to PAs, suggesting that VEGF might be associated with salivary gland cancer pathogenesis and aggressiveness. Fernández et al. examined the expression of VEGF protein in 66 salivary gland carcinomas and elucidated the relation between VEGF and clinicopathological parameters (54). VEGF expression was seen in 41 tumors (62%) and was correlated with lymph node metastasis (p < 0.005), clinical stage (p < 0.02), cause-specific survival (p < 0.01), and local failure-free survival (p < 0.02). They suggested that VEGF can contribute to the progression of salivary gland carcinomas and seems to be associated with neck node metastasis, worse survival and poor local control of the disease. Soares et al. investigated the angiogenic switch during the malignant transformation of PA into Ca-ex-PA, including 10 PAs, 8 early Ca-ex-PAs, and 8 advanced Ca-ex-Pas, and proved that angiogenesis was gradually but significantly increased from PAs to widely invasive Ca-ex-Pas (55). All of the above reports suggest that VEGF is involved in malignant conversion in salivary gland cancers.

**Figure 4:**
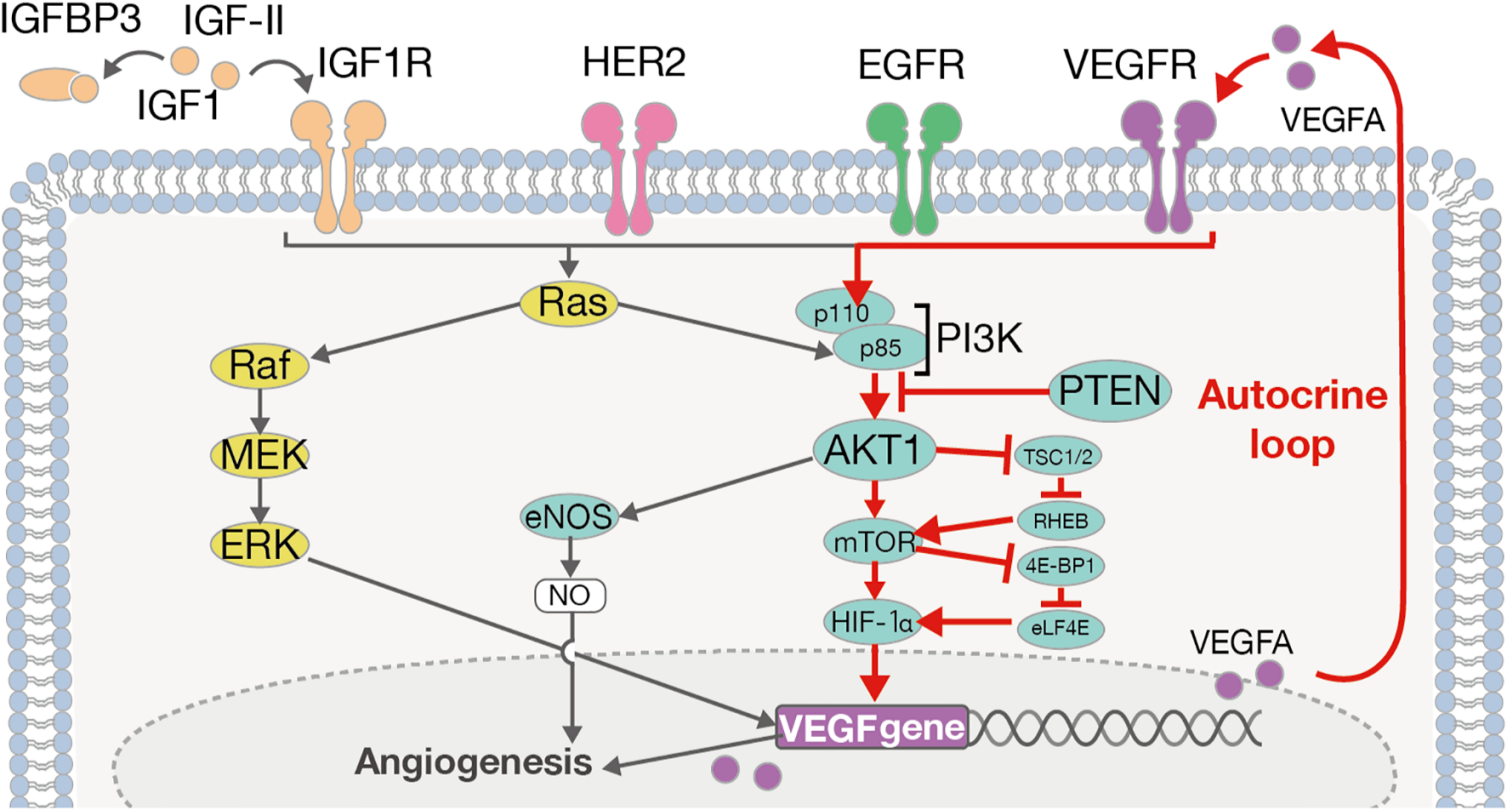
The ERBB2/VEGFA-AKT-PI3K-VEGFR signaling pathway and autocrine VEGFA signaling loop. VEGFA induces its autonomous proliferation *via* the pathway of AKT/PI3K/VEGFA, or that of Ras/Raf/MEK/ERK, identified as an “autocrine VEGFA signaling loop” in many other cancers.

Furthermore, *VEGFA* is also known to play a key role in the PI3K/AKT pathway (49) (**Figure 4**). PI3K activation occurs *via RAS* mutation, loss of *PTEN*, or by increased expression of growth factor receptors such as *ERBB2*, *EGFR*, *IGF1R*, and *VEGFA*. On the other hand, the activation of the PI3K/AKT pathway in tumor cells can promote the secretion of *VEGFA*, both by hypoxia-inducible factor 1 (*HIF-1α*)-dependent and -independent mechanisms (49). That is, *VEGFA* causes autonomous proliferation itself *via* the AKT/PI3K/VEGFA pathway, which has previously been identified as an “autocrine VEGFA signaling loop” in numerous other cancers (56). In the current study, we observed high levels of *VEGFA*, *AKT1*, and *IGF1R* expression, which indicates the possibility of the presence of an “autocrine VEGFA signaling loop” in SDCs. Furthermore, in the Ca-ex-PA SDCs, the expression levels of *VEGFA*, *ERBB2* and *IGF1R* were increased while that of *PTEN* was decreased. These results suggested that the AKT/PI3K/VEGFA pathway could be activated more aggressively in Ca-ex-PA than in *de novo* SDCs, and further promote angiogenesis.

**Figure 5:**
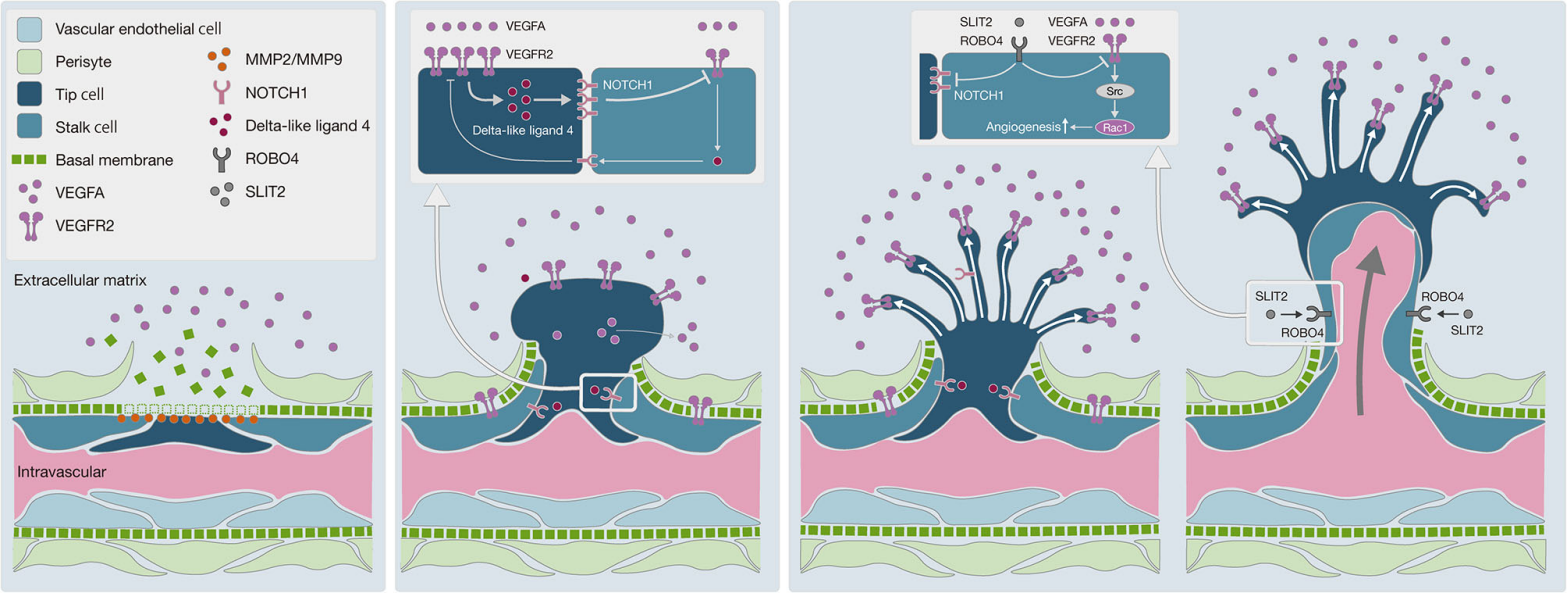
Schematic diagram of angiogenesis mechanism. **(A)** Degradation of basal membrane: MMPs, including MMP2 and MMP9, promote basal membrane degradation, subsequently vascular endothelial cells can migrate into the extracellular matrix. **(B)** Pericyte migration to the extracellular matrix: the VEGF-NOTCH feedback loop is involved in selective transformation of vascular endothelial cells into tip cells or stalk cells. Persistent VEGFA exposure causes abnormal acceleration of the VEGF-NOTCH feedback loop. **(C)** Negative control on angiogenesis by the SLIT2-ROBO4 system: SLIT2 inhibits VEGFR2 and NOTCH1 in tip cells and stalk cells *via* ROBO4. SLIT2-ROBO4 plays the role of “brake” for the VEGF-NOTCH feedback loop.

With regard to the mechanism underlying angiogenesis, recent studies have provided tremendous insights into fundamental aspects of angiogenesis that have led to a mechanistic model of vessel branching (57, 58) (**Figure 5**). Under quiescent normal conditions, the basal membrane is located between endothelial cells (ECs) and mural cells, preventing resident ECs from leaving their position relative to the coating of mural cells. MMPs, including MMP2 and MMP9, which are expressed by many cell types, including fibroblasts, keratinocytes, and ECs, degrade the basal membrane in cooperation with VEGFA in the extracellular matrix to promote EC migration and generate angiogenesis (59). In this study, we observed high levels of *MMP2* and *MMP9* expression in SDCs in general, suggesting greater EC migration into the extracellular matrix and the promotion of angiogenesis.

Moreover, ECs transform into tip cells and stalk cells. Tip cells lead to the formation of new sprouts and explore whether the environment is suitable for angiogenesis, while stalk cells adjacent to tip cells form a lumen and support tip cells in the elongation of the sprouts. Both cells play critical roles in concert with each other. Bentley et al. reported the mechanism of the VEGFR-Dll4 (delta like canonical Notch ligand 4)-Notch-VEGFR feedback loop between tip cells and stalk cells, which regulates the selection of tip cells and stalk cells (60). As this feedback loop is repeated, the differentiation of ECs into tip cells and stalk cells is promoted, resulting in hyper-vascularization. To regulate this feedback loop, SLIT2-ROBO4 (roundabout guidance receptor 4) works as a “brake”, with SLIT2 inhibiting VEGFR and NOTCH1 through the ROBO4 receptor on the cell membrane of tip cells and stalk cells (57, 61). In Ca-ex-PA SDCs, we observed a higher level of *VEGFA* and a lower level of *SLIT2* expression, which indicates that the regulation of the VEGFR-Dll4-Notch-VEGFR feedback loop by SLIT2/ROBO4 might have been out of control, resulting in hyper-activation of the feedback loop. These results suggested that uncontrolled angiogenesis could be promoted in SDCs, particularly in Ca-ex-PA type.

## Limitations

There are some limitations to this study. The first limitation is that the sample size is small due to the rarity of SDC. Secondly, we did not provide *in vitro* result showing the possible relationship between the gene expression and the carcinogenesis of SDC, because there is no established cell line representing SDC. Although the combination of *in vivo* and in silico methods in this study can reveal the possible involvement of gene expression in the carcinogenesis, further studies including *in vitro* model will be necessary.

## Conclusion

Our research indicated that the new classification of SDCs based on immune-phenotype appropriately reflects each gene expression profile. Our analysis of the onset classification of SDCs showed that there were significant differences in the expression of some genes related to angiogenesis and the AKT/PI3K signaling pathway. *VEGFA*, in particular, appears to be important in the transformation from PA to Ca-ex-PA and the aggressive behavior of SDCs, and affords a new target for approaches to the treatment of SDC.

## Data Availability Statement

The original contributions presented in the study are included in the article/**Supplementary Material**. Further inquiries can be directed to the corresponding author.

## Ethics Statement

The present study (016-0029) was approved by the Institutional Ethics Review Board of the Ethics Committee of our institution, and informed consent was obtained from each patient.

## Author Contributions

The finalized blocks were sliced into 4 mm-thick sections and mounted on glass slides. To check the histopathological diagnosis and adequacy of tissue sampling, a section from each microarray was stained with hematoxylin and eosin and examined by two expert pathologists (TS, KH). TS, SK, and AH conceived the study and provided suggestion and supervision of the study. TS, AH, SK, TM, SY, and NT collected and registered the data. TS, KH, and YM checked the histopathological diagnosis. TS and YH constructed each microarray and conducted immunostaining and dual color *in situ* hybridization. TS and MS conducted qRT-PCR. TS and MS contributed to data analysis. All authors contributed to the article and approved the submitted version.

## Acknowledgments

This study was supported by AMED 17lk0201074h0001 and JSPS KAKENHI Grant Number 18K16871 and 18KK0444.

## Conflict of Interest

The authors declare that the research was conducted in the absence of any commercial or financial relationships that could be construed as a potential conflict of interest.

## Supplementary Material

Supplementary Figure 1 OPLS-DA score map. OPLS-DA score map shows the two groups have been classified definitely by OPLS-DA.

Supplementary Figure 2 Variable Importance in Projection in OPLS-DA. Fourteen genes of VIP >1.5 were found, ordered as follows: *VEGFA*, *XBP1*, *PTEN*, *PYCARD*, TFF3, *MKI67*, *RASSF1*, *TP73*, *SLIT2*, *CDK2*, *ERBB2*, *ESR2*, *MMP2*, and *BRCA1*.

Supplementary Figure 3 Variable Importance by logistic regression model with L1 regularization. Nine genes, in the following order: *MKI67*, *AR*, *GSTP1*, *CTSD*, *PTEN*, *ID1*, *SFRP1*, *CSF1*, and *KRT18*, had non-zero coefficients, which could be sufficiently significant in this model.

Supplementary Figure 4 MeanDecreaseGini in RandomForest. *NOTCH1*, *CDKN1C*, *ID1*, *KRT8*, *RB1*, and *VEGFA* had high MeanDecreaseGini values for onset classification.
