# Supplementary figures and images for: Enhanced Angiogenesis in Salivary Duct Carcinoma Ex-Pleomorphic Adenoma
Source: Front Oncol. 2021 Feb 22;10:603717. doi: 10.3389/fonc.2020.603717 (PMC7937931; doi:10.3389/fonc.2020.603717)

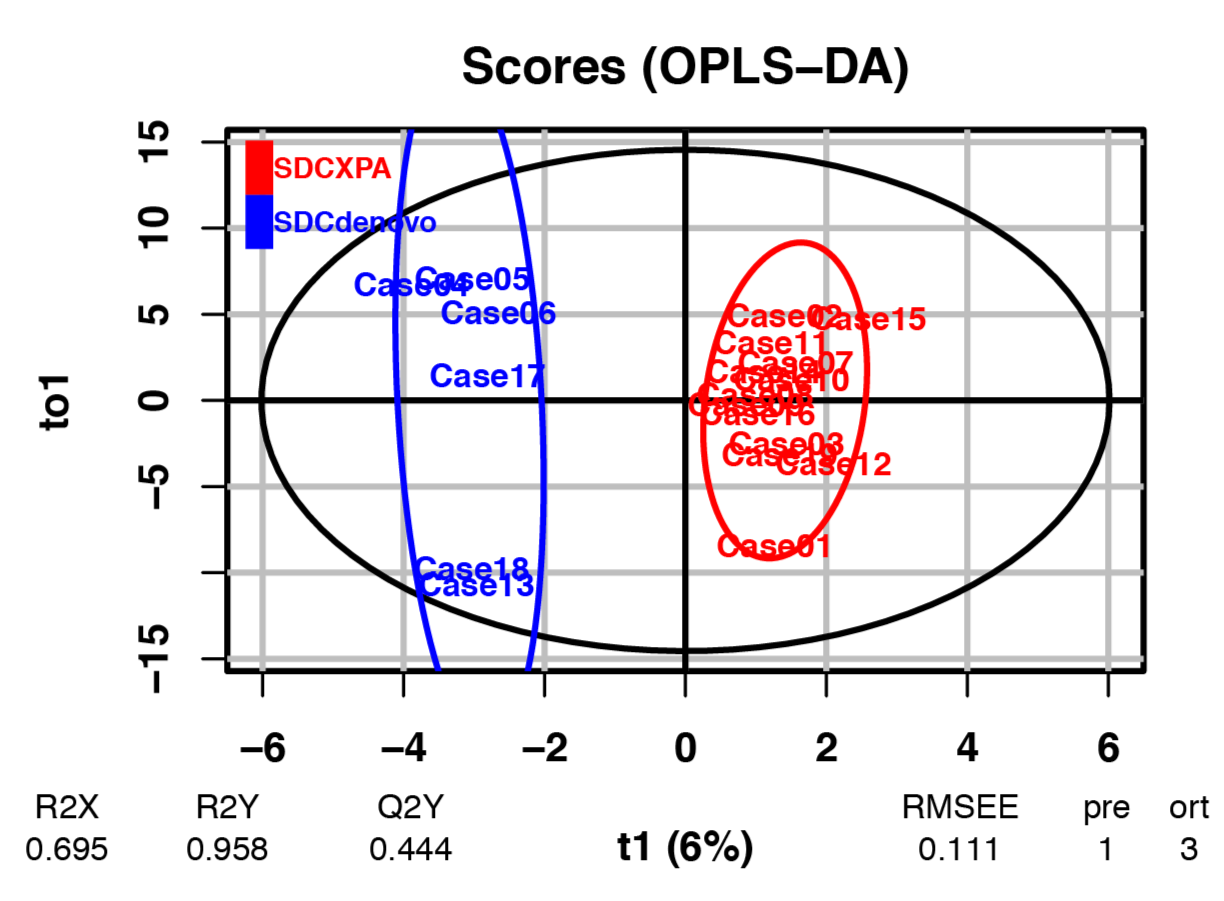

Supplement: Supplementary Figure 1 — OPLS-DA score map. OPLS-DA score map shows the two groups have been classified definitely by OPLS-DA. [file Image_1.tif]

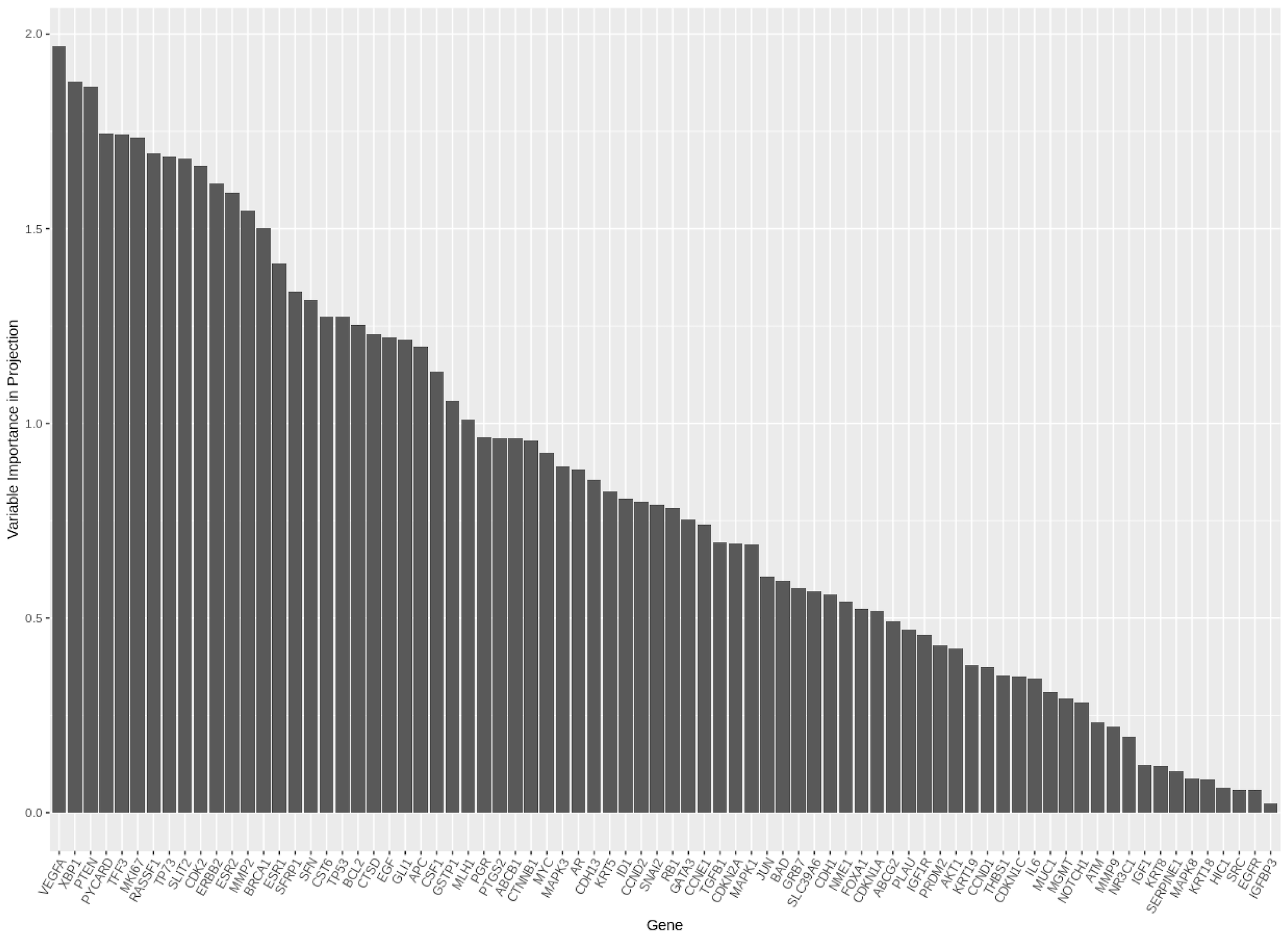

Supplement: Supplementary Figure 2 — Variable Importance in Projection in OPLS-DA. Fourteen genes of VIP >1.5 were found, ordered as follows: VEGFA, XBP1, PTEN, PYCARD, TFF3, MKI67, RASSF1, TP73, SLIT2, CDK2, ERBB2, ESR2, MMP2, and BRCA1. [file Image_2.tif]

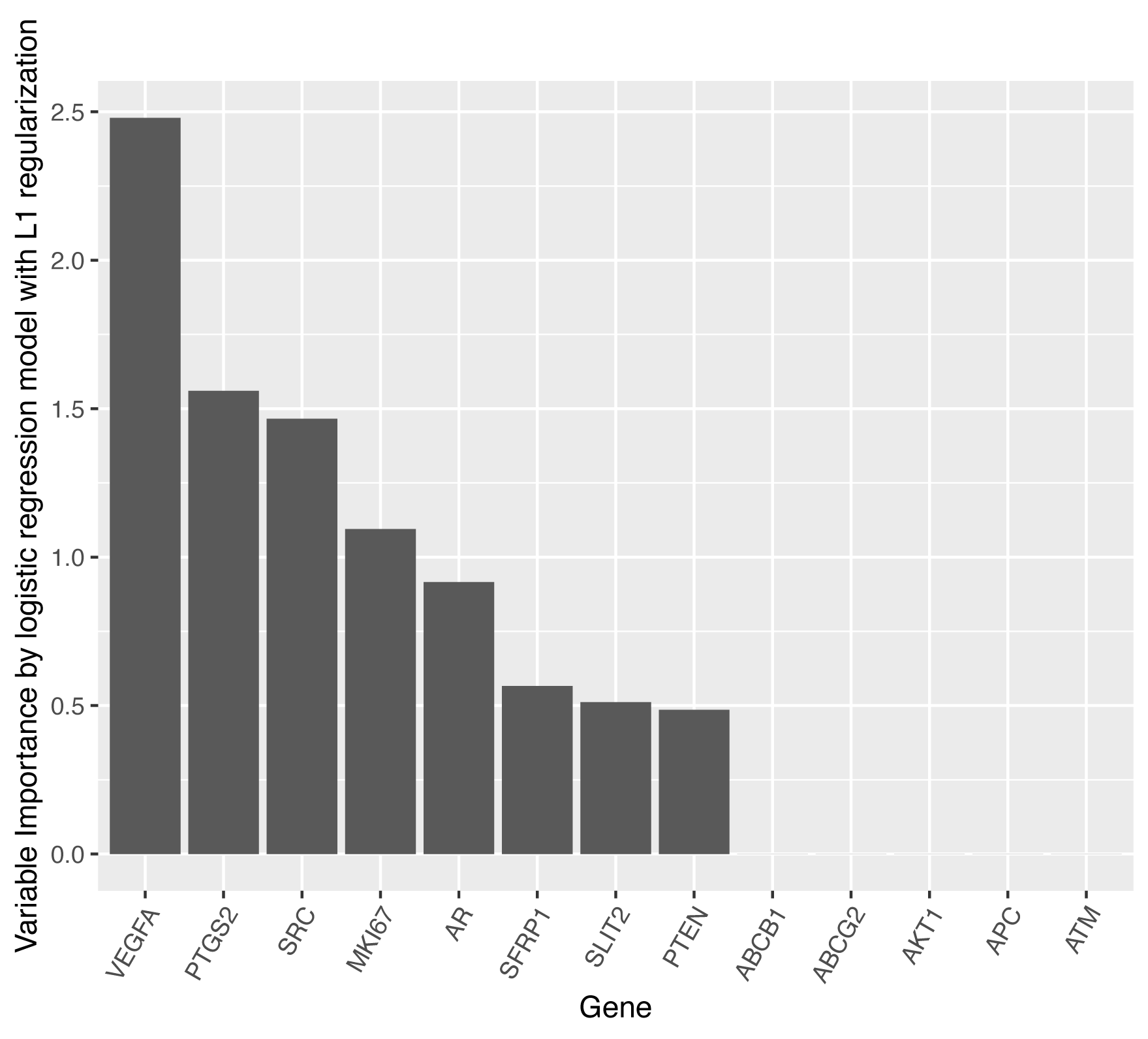

Supplement: Supplementary Figure 3 — Variable Importance by logistic regression model with L1 regularization. Nine genes, in the following order: MKI67, AR, GSTP1, CTSD, PTEN, ID1, SFRP1, CSF1, and KRT18, had non-zero coefficients, which could be sufficiently significant in this model. [file Image_3.tif]

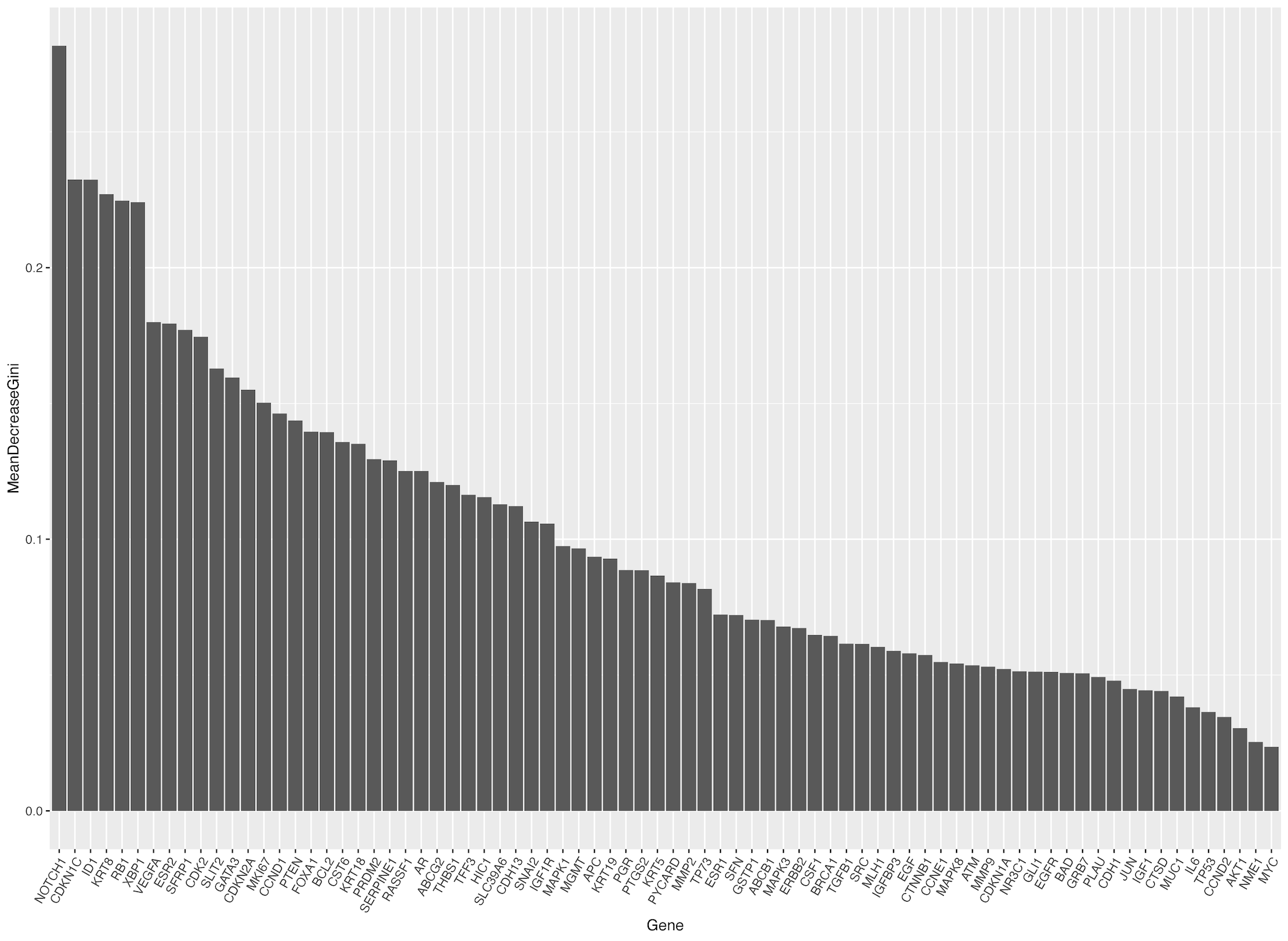

Supplement: Supplementary Figure 4 — MeanDecreaseGini in RandomForest. NOTCH1, CDKN1C, ID1, KRT8, RB1, and VEGFA had high MeanDecreaseGini values for onset classification. [file Image_4.tif]
